# Supplementary material for: Faster Walking Speeds Require Greater Activity from the Primary Motor Cortex in Older Adults Compared to Younger Adults
Source: Sensors (Basel). 2023 Aug 3;23(15):6921. doi: 10.3390/s23156921 (PMC10422240; doi:10.3390/s23156921)
Supplement: Supplementary file 1 [file sensors-23-06921-s001.zip › sensors-2444393-supplementary.pdf]

Table S1. Linear Mixed Effects Model evaluating the main effect of group (age) upon regional HbO2 Δ (%) and temporal-spatial gait outcomes.

Cortical region (HbO2; %)	Young Adults (n=17)	Old Adults (n=18)	Group (Sig.)
Left Prefrontal cortex	−11.5 [−25.1, 6.1]	3.1 [−8.3, 19.6]	$p = 0.001$*
Left Premotor cortex	−8.2 [−20.2, 1.9]	2.1 [−10.2, 20.8]	$p \leq 0.001$*
Left Supplementary motor area	−1.3 [−31.6, 7.9]	6.0 [−6.8, 12.0]	$p = 0.002$*
Left Primary motor cortex	0.3 [−21.4, 14.4]	9.3 [−3.6, 26.8]	$p = 0.002$*
Right Prefrontal cortex	−3.1 [−17.1, 8.5]	9.7 [−7.4, 22.5]	$p = 0.009$
Right Premotor cortex	−4.1 [−20.2, 14.1]	5.8 [1.6, 15.3]	$p = 0.010$
Right Supplementary motor area	−2.0 [−17.5, 9.7]	13.8 [3.4, 20.2]	$p \leq 0.001$*
Right Primary motor cortex	4.5 [−17.1, 20.4]	7.7 [−0.7, 21.2]	$p = 0.072$
**Temporal-spatial Gait**	**Young Adults (n=17)**	**Old Adults (n=18)**	**Group (Sig.)**
Mean			
Step time (s)	−0.048 [−0.054, −0.042]	−0.028 [−0.052, −0.012]	$p \leq 0.001$*
Stance time (s)	−0.038 [−0.050, −0.031]	−0.021 [−0.033, −0.007]	$p \leq 0.001$*
Swing time (s)	−0.057 [−0.066, −0.044]	−0.043 [−0.065, −0.022]	$p \leq 0.001$*
Step length (m)	0.029 [0.012, 0.040]	0.020 [0.009, 0.033]	$p = 0.001$*
Variability			
Step time (s)	−0.002 [−0.010, 0.001]	−0.008 [−0.016, 0.000]	$p = 0.024$
Stance time (s)	−0.002 [−0.010, 0.000]	−0.009 [−0.025, 0.000]	$p = 0.004$*
Swing time (s)	−0.002 [−0.009, 0.000]	−0.005 [−0.025, 0.000]	$p = 0.012$
Step length (m)	−0.009 [−0.016, −0.006]	−0.011 [−0.018, −0.003]	$p = 0.168$

Δ; fast – preferred. Data are presented group median [25th, 75th percentile]. * denotes significant differences for regional HbO2 and temporal-spatial gait outcomes (Bonferroni correction, $p \leq 0.006$).

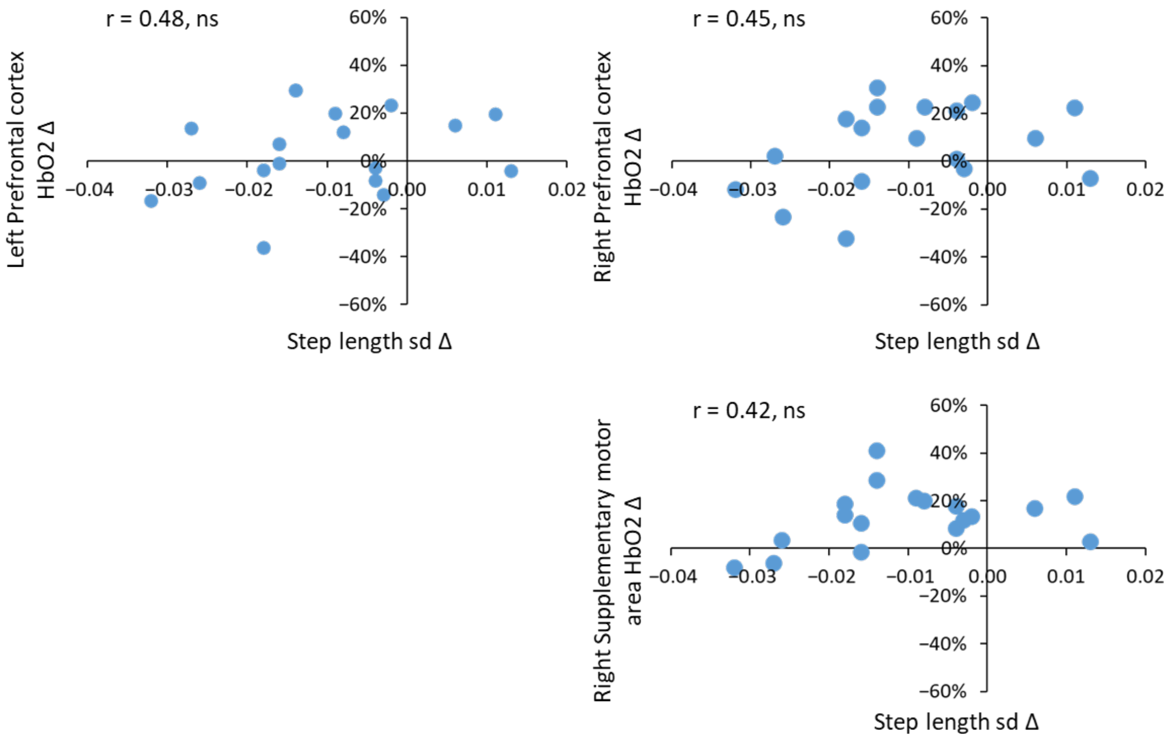

Figure S1. Partial correlations of moderate strength between step length variability (m) and regional HbO2 (%) for older adults. x axis: Δ step length (fast – preferred), y axis Δ regional HbO2 (fast – preferred). Positive values indicate fast > preferred, negative values indicate fast < preferred. ns denotes non-significant.
